# The relationship between parental psychological control, cyberbullying victimization, and nonsuicidal self-injury among boarding school adolescents

**DOI:** 10.3389/fpsyt.2025.1695119

**Published:** 2026-01-14

**Authors:** Chang Wei, Lanping Wang, Huixing Lu, Jingjing Li

**Affiliations:** 1 School of Arts and Sciences, Guangzhou Maritime University, Guangzhou, China; 2 Nansha Experimental School, High School Affiliated to Guangzhou University, Guangzhou, China; 3 Research Center for Rural Educational and Cultural Development of Key Research Base of Humanities and Social Sciences in Hubei Province, School of Education, Hubei University of Science and Technology, Xianning, China; 4 School of Health Management, Guangzhou Medical University, Guangzhou, China

**Keywords:** adolescents, boarding school, cyberbullying victimization, non-suicidal self-injury, parental psychological control

## Abstract

**Objective:**

This study aims to investigate the relationship between parental psychological control and nonsuicidal self-injury among adolescents in boarding schools and to examine the mediating role of cyberbullying victimization in this relationship.

**Methods:**

This study included 378 adolescents from boarding schools and utilized the Parental Psychological Control Scale, the Cyberbullying Victimization Scale, and the Nonsuicidal Self-injury Scale.

**Results:**

Parental psychological control was significantly positively correlated with nonsuicidal self-injury among adolescents in boarding schools. Cyberbullying victimization mediated the relationship between parental psychological control and nonsuicidal self-injury (indirect effect = 0.017, SE = 0.008 [95% CI = 0.003, 0.034]).

**Conclusions:**

This finding highlights that nonsuicidal self-injury among adolescents in boarding schools results from the combined effect of distal family factors (e.g., parental psychological control) and proximal cyber stress (e.g., cyberbullying victimization), providing a reference for future targeted interventions.

Nonsuicidal self-injury is defined as the deliberate and intentional harm to one’s own body tissue without the intent to die (1). Adolescence is a peak period for nonsuicidal self-injury. A meta-analysis spanning 41 countries revealed that the lifetime prevalence of nonsuicidal self-injury among adolescents is as high as 16.9% (2). Nonsuicidal self-injury is not only a significant risk factor for adolescent mental health problems but also a strong predictor of suicide (3–5). Thus, identifying the risk factors for nonsuicidal self-injury among adolescents is of paramount importance.

Previous studies have predominantly focused on general adolescent samples (6, 7), with insufficient attention to adolescents in boarding schools. The unique ecological environment of boarding schools poses numerous challenges for these adolescents in their adjustment process. Therefore, the present study focuses on adolescents in boarding schools to explore the relationship between parental psychological control and nonsuicidal self-injury, as well as the mediating role of cyberbullying victimization, thereby uncovering the underlying pathways of risk transmission within this specific population.

## Parental psychological control and nonsuicidal self-injury

Parental psychological control refers to parental behaviors that are intrusive and manipulative of children’s thoughts, feelings, and attachment to parents (8). According to ecological systems theory, the family, as a key microsystem in adolescent development, exerts a direct and lasting influence on adolescents (9). Previous research has revealed a close association between parental psychological control and adolescent problem behaviors (10, 11). According to the self-determination theory (12), parental psychological control may thwart adolescents’ basic psychological needs, particularly the need for autonomy. When these needs are frustrated, adolescents are likely to experience negative emotions, which in turn may increase the risk of nonsuicidal self-injury (13–15). Moreover, existing research has confirmed that parental psychological control is a significant risk factor for adolescent nonsuicidal self-injury (16, 17). For example, in a sample of 1,006 Chinese adolescents, Huang et al. (17) found that parental psychological control was significantly positively associated with nonsuicidal self-injury. Similarly, Guo et al. (16) found that parental psychological control was a risk factor for nonsuicidal self-injury among adolescents. Grounded in ecological systems theory (9) and self-determination theory (12), and supported by empirical evidence, the following hypothesis is proposed:

Hypothesis 1: Parental psychological control is significantly positively correlated with nonsuicidal self-injury.

## Cyberbullying victimization as a potential mediator

Cyberbullying refers to the actions of an individual or a group using online platforms to repeatedly send hostile or aggressive messages to others with the intent to cause harm or discomfort (7, 18, 19). There is evidence supporting the mediating role of cyberbullying victimization between parental psychological control and adolescent nonsuicidal self-injury. First, cyberbullying victimization has been empirically identified as a significant risk factor for adolescent nonsuicidal self-injury (7, 20, 21). For example, in a study of 1,102 Chinese adolescents, Yu et al. (7) found that cyberbullying victimization was positively correlated with nonsuicidal self-injury. Second, previous research has confirmed the association between parental psychological control and cyberbullying victimization. For example, in a study of 2,445 Chinese adolescents, Ren et al. (22) found that parental psychological control was positively correlated with cyberbullying victimization. Moreover, according to the integrated theoretical model of the development and maintenance of nonsuicidal self-injury (23), parental psychological control leads to adolescents’ vulnerability in interpersonal interactions, thereby raising their risk of experiencing cyberbullying. To alleviate the distress caused by cyberbullying victimization, these adolescents may resort to nonsuicidal self-injury.

Additionally, existing research has confirmed that traditional bullying victimization plays a significant mediating role between parental psychological control and nonsuicidal self-injury among adolescents (24). With the increasing online nature of adolescents’ social activities, cyberbullying has become more prominent. Relevant studies have shown that the prevalence of cyberbullying victimization among children and adolescents ranges from 13.99% to 57.5% (25). Despite this, the mediating role of cyberbullying victimization in the relationship between parental psychological control and nonsuicidal self-injury has not been fully explored. The present study aims to examine this mediating role to gain a more comprehensive understanding of the underlying mechanisms of the relationship. Grounded in the integrated theoretical model of the development and maintenance of nonsuicidal self-injury (23) and supported by empirical evidence, the following hypothesis is proposed:

Hypothesis 2: Cyberbullying victimization will mediate the association between parental psychological control and nonsuicidal self-injury.

## The present study

**Figure 1:**
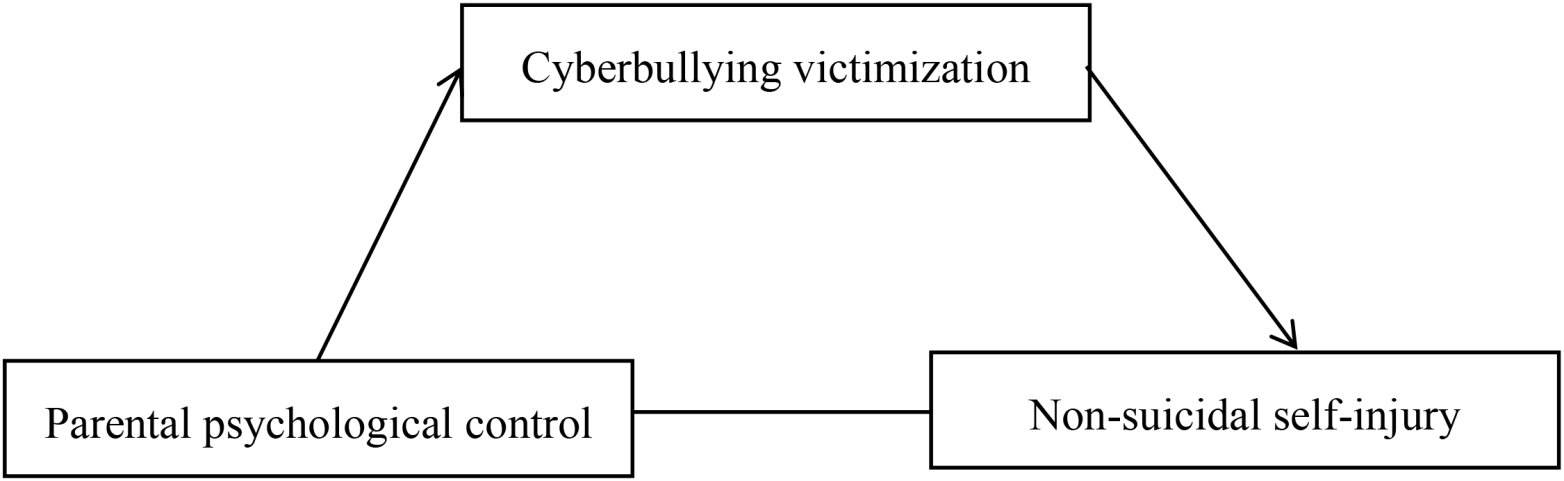
Proposed mediation model.

The present study examined the relationship between parental psychological control and nonsuicidal self-injury (Hypothesis 1) and further tested whether cyberbullying victimization mediates the relationship between parental psychological control and nonsuicidal self-injury (Hypothesis 2). **Figure 1** presents the proposed model.

## Method

### Participants

A total of 378 Chinese adolescents from boarding schools were included in this study, all of whom were junior high school students. Among them, 198 were boys (52.4%) and 180 were girls (47.6%). In terms of place of residence, 195 participants were from rural areas (51.6%) and 183 were from urban areas (48.4%). Regarding the left-behind status, 145 were left-behind children (38.4%) and 233 were not left-behind children (61.6%). The adolescents ranged in age from 12 to 15 years old (*M*_age_ = 12.97; SD = 0.51).

### Procedure

This study was approved by the Academic Ethics Review Board of the author’s university. Informed consent was obtained from participants and their parents/guardians prior to data collection. All participants were informed that their participation was completely voluntary and that they had the right to withdraw at any time.

### Measures

#### Parental psychological control

Parental psychological control was measured using the Parental Control Scale (26). This scale includes 10 items (e.g., “My parents often try to change the way I think about or feel about things.”) rated on a five-point scale (1 = never like this to 5 = always like this). The measure is calculated using the average score of 10 items, with higher scores indicating a higher level of parental psychological control. In this study, Cronbach’s alpha was 0.94.

#### Cyberbullying victimization

Cyberbullying victimization was measured using the Cyberbullying Subscale of the Revised Chinese Version of the Delaware Bullying Victimization Scale (27). This scale includes four items (e.g., “A student sent me a mean or hurtful message through email, text message, WeChat, QQ, or a similar electronic communication method.”) rated on a six-point scale (1 = never to 6 = every day). The measure is calculated as the average score of the four items, with higher scores indicating a higher level of cyberbullying victimization. In this study, Cronbach’s alpha was 0.86.

#### Nonsuicidal self-injury

Nonsuicidal self-injury was measured by selecting 12 nonsuicidal self-injury behaviors (28) from the Deliberate Self-Harm Inventory Scale (29). This scale includes 12 items (e.g., “biting yourself”) rated on a six-point scale (0 = never to 5 = 5 or more times). The measure is calculated using the average score of the 12 items, with higher scores indicating a higher level of nonsuicidal self-injury. In this study, Cronbach’s alpha was 0.89.

#### Control variables

According to existing research, gender and age impact nonsuicidal self-injury (30, 31). Therefore, gender and age were included in our analysis as control variables.

### Statistical analyses

We used SPSS 27.0 to generate descriptive statistics. We adopted Model 4 of the PROCESS for SPSS to examine whether cyberbullying victimization mediated the association between parental psychological control and nonsuicidal self-injury. Given that the missing data constituted less than 1%, a normality test was performed. The results revealed a skewed distribution of the data; consequently, median imputation was used to address the missing values.

## Results

### Preliminary analyses

**Table 1** shows the means, standard deviations, and correlation coefficients for all study variables. Parental psychological control was positively associated with both cyberbullying victimization and nonsuicidal self-injury. In addition, cyberbullying victimization was positively associated with nonsuicidal self-injury.

**Table 1:** Descriptive statistics and correlations for all variables.

Variable	1	2	3	4	5
1. Gender	1.00				
2. Age	0.11^*^	1.00			
3. Parental psychological control	− 0.06	0.02	1.00		
4. Cyberbullying victimization	0.02	0.04	0.12^*^	1.00	
5. Nonsuicidal self-injury	− 0.13^*^	0.08	0.23^***^	0.33^***^	
Mean	0.52	12.97	2.41	1.07	0.15
SD	0.50	0.51	0.96	0.32	0.44

Gender was dummy coded as 1 = boy, 0 = girl.

^*^p < 0.05; ^***^p < 0.001.

### Mediation effect of cyberbullying victimization

**Figure 2:**
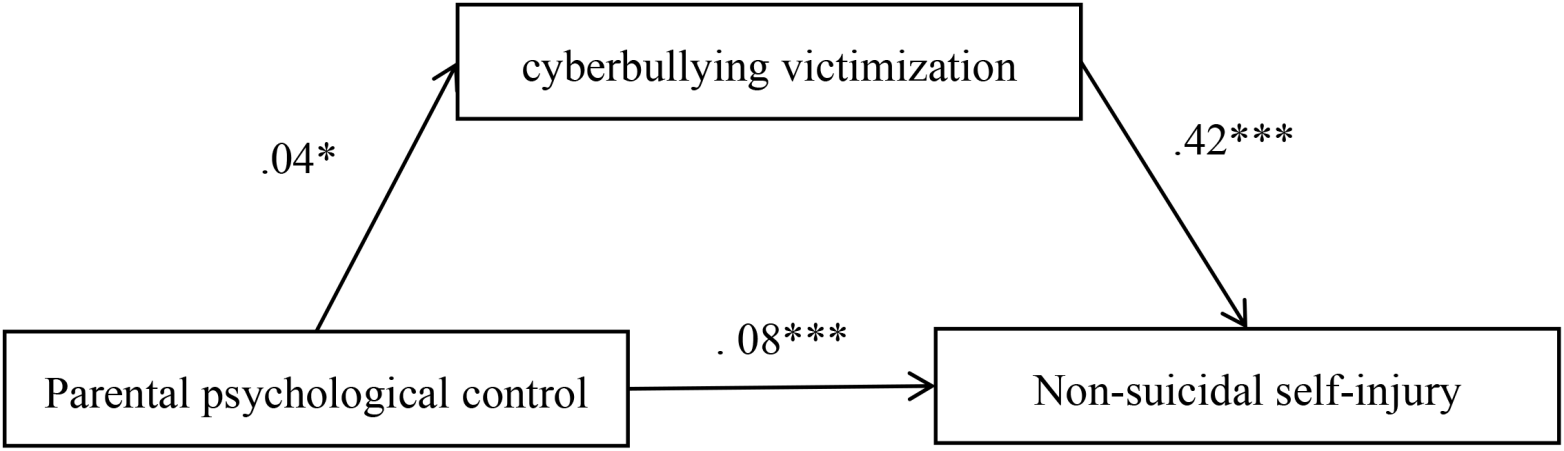
Mediational analysis of cyberbullying victimization in the association between parental psychological control and nonsuicidal self-injury. ^*^p < 0.05; ^***^p < 0.001.

**Figure 2** shows the results obtained from the mediation model. After controlling for gender and age, parental psychological control positively predicted cyberbullying victimization (*b* = 0.04, *β* = 0.12, SE(*b*) = 0.02, *p* < 0.05), which in turn positively predicted nonsuicidal self-injury (*b* = 0.42, *β* = 0.31, SE(*b*) = 0.07, *p* < 0.001). The bias-corrected percentile bootstrap method showed a significant mediating effect of cyberbullying victimization in the relationship between parental psychological control and nonsuicidal self-injury (indirect effect = 0.017, SE = 0.008 [95% CI = 0.003, 0.034]). Details shown in **Table 2**.

**Table 2:** Mediating effect of cyberbullying victimization in the relationship between parental psychological control and nonsuicidal self-injury.

	Model 1 (CV)				Model 2 (NSSI)			
	*b*	*β*	*t (b)*	95% CI (*b*)	*b*	*β*	*t (b)*	95% CI (*b*)
Gender	0.01	0.02	0.43	[− 0.05, 0.08]	− 0.12	− 0.14	− 2.86^**^	[− 0.20, − 0.04]
Age	0.03	0.04	0.81	[− 0.04, 0.09]	0.08	0.09	1.89	[0.00, 0.16]
PPC	0.04	0.12	2.38^*^	[0.01, 0.07]	0.08	0.19	3.90^***^	[0.04, 0.13]
CV					0.42	0.31	6.48^***^	[0.29, 0.55]
*R* ^2^	0.02				0.17			
*F*	2.13				19.15^***^			

Sample size *N* = 378. Gender was dummy coded as 1 = boy, 0 = girl.

*PPC*, parental psychological control; *CV*, cyberbullying victimization; *NSSI*, nonsuicidal self-injury.

^*^p < 0.05; ^**^p < 0.01; ^***^p < 0.001.

## Discussion

Based on the ecological systems theory (9), the self-determination theory (12), and the integrated theoretical model of the development and maintenance of nonsuicidal self-injury (23), this study investigated the relationship between parental psychological control and nonsuicidal self-injury among adolescents in boarding schools and examined the mediating role of cyberbullying victimization in this relationship. The results indicated that parental psychological control was significantly positively correlated with nonsuicidal self-injury, and cyberbullying victimization partially mediated the relationship between parental psychological control and nonsuicidal self-injury.

### Relationship between parental psychological control and nonsuicidal self-injury

The findings supported Hypothesis 1: this study found a significant positive correlation between parental psychological control and nonsuicidal self-injury among adolescents in boarding schools. This result is consistent with previous research findings, further substantiating that parental psychological control is a key risk factor for nonsuicidal self-injury in adolescents (16, 17). Additionally, this study focused on adolescents in boarding schools. According to self-determination theory (12), excessive parental psychological control may suppress the basic psychological needs of adolescents in boarding schools (such as the need for autonomy), thereby increasing the risk of nonsuicidal self-injury (15). The study results suggest that educators in boarding schools need to pay particular attention to the psychological interactions between students and their parents, as well as the potential impact of these interactions on students’ nonsuicidal self-injury behaviors.

### The mediating role of cyberbullying victimization

The findings supported Hypothesis 2, which posits that cyberbullying victimization mediates the association between parental psychological control and nonsuicidal self-injury. This indicates that cyberbullying victimization is a key bridge linking parental psychological control to nonsuicidal self-injury among adolescents in boarding schools. Adolescents experiencing parental psychological control may develop avoidance motivation, making them more susceptible to cyberbullying (32). Additionally, the negative emotional experiences triggered by cyberbullying victimization may prompt adolescents to engage in nonsuicidal self-injury as a way of coping with these emotions (7).

Although the effect size of the mediating effect in this study is relatively small, its significance cannot be overlooked. This study empirically reveals a complete chain of risk transmission from “family risk” (parental psychological control) through “cyber risk” (cyberbullying victimization) to “maladaptive behavior” (nonsuicidal self-injury), providing important evidence for understanding nonsuicidal self-injury behavior.

This study not only empirically revealed the chain of effects —”parental psychological control→cyberbullying victimization→nonsuicidal self-injury”—but also strengthened the empirical basis of the integrated theoretical model of the development and maintenance of nonsuicidal self-injury (23) for the specific group of boarding school adolescents. Additionally, these findings provide clear direction for interventions targeting nonsuicidal self-injury among adolescents in boarding schools. The results suggest that educators in boarding schools should enhance supervision of adolescents’ online behavior and actively foster a healthy online environment to reduce the occurrence of cyberbullying. Meanwhile, by conducting mental health education courses, educators can teach students skills in stress management and emotion regulation, thereby effectively reducing the risk of nonsuicidal self-injury in this population.

### Limitations and future directions

This study had several limitations. First, reliance solely on adolescents’ self-reports for all assessments may have led to inflated associations due to common method bias. Future research should include data from multiple sources, such as parents and class teachers, to reduce this bias. Second, given the cross-sectional nature of this study, causal inferences regarding the relationships among the variables cannot be made. Future research should consider adopting a longitudinal design to investigate further the relationships among parental psychological control, cyberbullying victimization, and nonsuicidal self-injury. Third, due to the significant deviation from the Hardy–Weinberg equilibrium in the genotype distribution data, this study was unable to examine the moderating effect of the 5-HTR2A gene rs6313 polymorphism as initially planned. Future research needs to explore the moderating role of genetic factors to gain a more comprehensive understanding of the relationship between parental psychological control, cyberbullying victimization, and nonsuicidal self-injury among adolescents. Fourth, although this study emphasizes the mediating role of cyberbullying victimization, the reported mediating effect size is small, suggesting that other stronger mediating variables between parental psychological control and nonsuicidal self-injury may not have been included in the model. For example, basic psychological needs (15) and depression (13) may also be important mediating variables. Future research should consider these alternative or parallel mechanisms to achieve a more comprehensive understanding of this relationship.

## Conclusions

This study explores the association between parental psychological control and nonsuicidal self-injury among adolescents in boarding schools, as well as the role of cyberbullying victimization in this relationship. The results highlight the mediating effect of cyberbullying victimization between parental psychological control and nonsuicidal self-injury. These findings not only enhance our understanding of the etiology of nonsuicidal self-injury but also provide references for future intervention strategies.

## Data Availability

The original contributions presented in the study are included in the article/supplementary material. Further inquiries can be directed to the corresponding author.

## References

[1] Nock MK . Self-injury. Annu Rev Clin Psychol. (2010) 6:339–63. doi: 10.1146/annurev.clinpsy.121208.13125 20192787

[2] Gillies D Christou MA Dixon AC Featherston OJ Rapti I Garcia-Anguita A . Prevalence and characteristics of self-harm in adolescents: Meta-analyses of community-based studies 1990–2015. J Am Acad Child Adolesc Psychiatry. (2018) 57:733–41. doi: 10.1016/j.jaac.2018.06.018, PMID: 30274648

[3] Calvo N García-González S Perez-Galbarro C Regales-Peco C Lugo-Marin J Ramos-Quiroga JA . Psychotherapeutic interventions specifically developed for NSSI in adolescence: A systematic review. Eur Neuropsychophar-macol. (2022) 58:86–98. doi: 10.1016/j.euroneuro.2022.02.009, PMID: 35325633

[4] Lei H Xiong J Rao Y Zhu T Zhang X . Relationships among self-esteem, depression and self-injury in adolescents: a longitudinal study. Front Public Health. (2024) 15:1406283. doi: 10.3389/fpubh.2024.1406283, PMID: 38813433 PMC11135207

[5] Mars B Heron J Klonsky ED Moran P O’Connor RC Tilling K . Predictors of future suicide attempt among adolescents with suicidal thoughts or non-suicidal self-harm: A population-based birth cohort study. Lancet Psychiat 6. (2019) 6:327–37. doi: 10.1016/s2215-0366(19)30030-6, PMID: 30879972 PMC6494973

[6] Wen Y . *The relationship beteen parental psychologyical control, bullied experience and adolescent NSSI: The moderating role of self-compassion* (published master’s thesis). JInan: Shandong Norm Univ (2024). doi: 10.27280/d.cnki.gsdsu.2024.000409

[7] Yu. C Xie Q Lin S Liang Y Wang G Nie Y . Cyberbullying victimization and non-suicidal self-injurious behavior among chinese adolescents: school engagement as a mediator and sensation seeking as a moderator. Front Psychol. (2020) 11:572521. doi: 10.3389/fpsyg.2020.572521, PMID: 33250816 PMC7674837

[8] Barber BK Harmon EL . Violating the self: parental psychological control of children and adolescents. In: Barber BK , editor. Intrusive parenting: how psychological control affects children and adolescents. American Psychological Association, Washington, DC (2002).

[9] Bronfenbrenner U . The ecology of human development: Experiments by nature and design. Cambridge, MA: Harvard University Press (1979).

[10] Deng L Xiong Y Yang M Li B . The mediating mechanism of parental conflict, parental control and high school students’ internet addiction: A longitudinal study. Chin J Special Edu. (2020) 242:89–96. doi: 10.3969/j.issn.1007-3728.2020.08.015

[11] Tian Y Yu C Lin S Lu J Liu Y Zhang W . Parental psychological control and adolescent aggressive behavior: deviant peer affiliation as a mediator and school connectedness as a moderator. Front Psychol. (2019) 10:358. doi: 10.3389/fpsyg.2019.00358, PMID: 30846957 PMC6393334

[12] Deci EL Ryan RM . The “what” and “why” of goal pursuits: Human needs and the self-determination of behavior. Psychol Inq. (2000) 11:227–68. doi: 10.1207/S15327965PLI1104_01

[13] Li M Wang H Li J Deng Y Yu C . Peer victimization, depression, and non-suicidal self-injury among Chinese adolescents: the moderating role of the 5-HTR2A gene rs6313 polymorphism. Child Adol Psych Men. (2022) 16:108. doi: 10.1186/s13034-022-00532-4, PMID: 36575481 PMC9795745

[14] Liu X Liu Y Zeng J . The longitudinal relationship between psychological need frustration and depressive symptoms in adolescents: the moderating role of psychological suzh. Stud Psychol Behav. (2025) 23:496–503. doi: 10.12139/j.1672-0628.2025.04.009

[15] Long J . *The relationship beteen parental psychologyical control, bullied experience and adolescent NSSI: The moderating role of self-compassion* (published master’s thesis). Xiangtan: Hunan Univ Sci Tech (2024). doi: 10.27738/d.cnki.ghnkd.2024.000139

[16] Guo J Gao Q Wu R Ying J You J . Parental psychological control, parent-related loneliness, depressive symptoms, and regulatory emotional self-efficacy: A moderated serial mediation model of non-suicidal self-injury. Arch Suicide Res 26. (2022) 26:1462–77. doi: 10.1080/13811118.2021.1922109, PMID: 34586982

[17] Huang J Zhang D Chen Y Yu C Zhen S Zhang W . parental psychological control and adolescent non-suicidal self-injury. Child Youth Serv Rev. (2022) 136:106417. doi: 10.1016/j.childyouth.2022.106417

[18] Li Q . Bullying in the new playground: research into cyberbullying and cybervictimization. Australas J Educ Tec. (2007) 232:435–54. doi: 10.14742/ajet.1245

[19] Smith PK Mahdavi J Carvalho M Fisher S Russell S Tippett N . Cyberbullying: its nature and impact in secondary school pupils. J Child Psychol Psychiatry. (2008) , 49:376–85. doi: 10.1111/j.1469-7610.2007.01846.x, PMID: 18363945

[20] Geng J Jiao L Pan S Liu Y Wang Y . The influence of cyberbullying victimization on adolescents’ engagement in non-suicidal self-injurious behavior: A longitudinal multi-mediation analysis. Child Abuse Negl. (2024) 161:107237. doi: 10.1016/j.chiabu.2024.107237, PMID: 39823767

[21] Wiguna T Minayati K Kaligis F Ismail R Wijaya E Murtani B . The effect of cyberbullying, abuse, and screen time on non-suicidal self-injury among adolescents during the pandemic: A perspective from the mediating role of stress. Front Psychiatry. (2021) 12:743329. doi: 10.3389/fpsyt.2021.743329, PMID: 34867535 PMC8632872

[22] Ren P Yang L Chen C Luo F . Parental control and adolescents’ bullying victimization: the moderating role of teacher support. Curr Psychol. (2023) 42:27952–64. doi: 10.1007/s12144-022-03864-8

[23] Nock MK . Why do people hurt themselves? New insights into the nature and functions of self-injury. Curr Dir. Psychol. (2009) 18:78–83. doi: 10.1111/j.1467-8721.2009.01613.x, PMID: 20161092 PMC2744421

[24] Zhang S Zhang Y . The impact of parental psychological control on non-suicidal self-injury: the role of campus bullying as a mediator. Chin J Dis Control Prew. (2019) 23:459–63. doi: 10.16462/j.cnki.zhjbkz.2019.04.018

[25] Zhu C Huang S Evans R Zhang W . Cyberbullying among adolescents and children: A comprehensive review of the global situation, risk factors, and preventive measures. Front Public Health. (2021) 9:634909. doi: 10.3389/fpubh.2021.634909, PMID: 33791270 PMC8006937

[26] Shek DTL . Perceived parental control and parent–child relational qualities in chinese adolescents in hong kong. Sex Roles. (2005) 53:635–46. doi: 10.1007/s11199-005-7730-7

[27] Xie J Wei Y Bear G . Revision of chinese version of delaware bullying victimization scale-student in adolescents. Chin. J Clin Psychol. (2018) 26:259–63. doi: 10.16128/j.cnki.1005-3611.2018.02.01

[28] Ren Y Lin M-P Liu Y-H Zhang X Wu JY-W Hu W-H . The mediating role of coping strategy in the association between family functioning and non-suicidal self-injury among Taiwanese adolescents. J Clin Psychol. (2018) 74:1246–57. doi: 10.1002/jclp.22587, PMID: 29355974

[29] Gratz KL . Measurement of deliberate self-harm: Preliminary data on the Deliberate Self-Harm Inventory. J Psychopathol Behav. (2001) 23:253–63. doi: 10.1023/A:1012779403943

[30] Rahman F Webb RT Wittkowski A . Risk factors for self-harm repetition in adolescents: A systematic review. Clin Psychol Rev. (2021) 88:102048. doi: 10.1016/j.cpr.2021.102048, PMID: 34119893

[31] Swannell SV Martin GE Page A Hasking P St John NJ . Prevalence of nonsuicidal self-injury in nonclinical samples: Systematic review, meta-analysis and meta-regression. Suicide Life Threat Behav. (2014) 44:273–303. doi: 10.1111/sltb.12070, PMID: 24422986

[32] Hsieh YP . Parental psychological control and adolescent cyberbullying victimization and perpetration: the mediating roles of avoidance motivation and revenge motivation. Asia Pac J Soc Work. (2020) 30:212–26. doi: 10.1080/02185385.2020.1776153

